# Atomic Layer Deposition of ZnO on Mesoporous Silica: Insights into Growth Behavior of ZnO via In-Situ Thermogravimetric Analysis

**DOI:** 10.3390/nano10050981

**Published:** 2020-05-20

**Authors:** Piyush Ingale, Kristian Knemeyer, Mar Piernavieja Hermida, Raoul Naumann d’Alnoncourt, Arne Thomas, Frank Rosowski

**Affiliations:** 1 BasCat—UniCat BASF JointLab, Technische Universität Berlin, Hardenbergstraße 36, 10623 Berlin, Germany; p.ingale@bascat.tu-berlin.de (P.I.); k.knemeyer@bascat.tu-berlin.de (K.K.); m.piernaviejahermida@tu-berlin.de (M.P.H.); frank.rosowski@basf.com (F.R.); 2 Institut für Chemie, Technische Universität Berlin, 10623 Berlin, Germany; arne.thomas@tu-berlin.de; 3 Process Research and Chemical Engineering, BASF SE, 67056 Ludwigshafen, Germany

**Keywords:** atomic layer deposition, ZnO, high surface area, diethylzinc, SiO_2_

## Abstract

ZnO is a remarkable material with many applications in electronics and catalysis. Atomic layer deposition (ALD) of ZnO on flat substrates is an industrially applied and well-known process. Various studies describe the growth of ZnO layers on flat substrates. However, the growth characteristics and reaction mechanisms of atomic layer deposition of ZnO on mesoporous powders have not been well studied. This study investigates the ZnO ALD process based on diethylzinc (DEZn) and water with silica powder as substrate. In-situ thermogravimetric analysis gives direct access to the growth rates and reaction mechanisms of this process. Ex-situ analytics, e.g., N_2_ sorption analysis, XRD, XRF, HRTEM, and STEM-EDX mapping, confirm deposition of homogenous and thin films of ZnO on SiO_2_. In summary, this study offers new insights into the fundamentals of an ALD process on high surface area powders.

## 1. Introduction

ZnO with its unique electronic and optical properties is truly a multi-functional material with applications in optoelectronics [1], sensors [2], photovoltaics [3], and catalysis [4]. ZnO is a wide bandgap semiconductor (e.g., 3.37 eV) with a large exciton binding energy (60 meV) [5]. ZnO has been investigated as support or promoter in various catalytic processes such as reverse water gas shift [6], methanol synthesis [7], alkane hydrogenation [8], and dehydrogenation [9]. ZnO offers an interesting choice as support material due to improved performance of catalyst systems such as Cu/ZnO/Al_2_O_3_. The interaction between Cu and ZnO leads to an increase in intrinsic activity of Cu-based methanol synthesis catalysts, which is often related to strong metal support interactions [10]. A variety of methods have been implemented in synthesis of ZnO structures such as precipitation [11] and hydrothermal synthesis [12] without any significant success in the development of stable high surface area ZnO.

Atomic layer deposition (ALD) is a thin film deposition technique widely applied in the semiconductor industry, which relies on two sequential reactions of vapor phase precursor and reactant to surface terminal groups [13]. ALD is advantageous for coating powders in the field of catalyst design due to its ability to ensure uniform coatings with high conformity [14]. Over the years, ALD has been used to deposit thin layers of various metals and their oxides such as Al_2_O_3_ [15], ZnO [16], and TiO_2_ [17]. ALD of ZnO is the second most widely investigated process after ALD of Al_2_O_3_. The first report on ALD of ZnO was published in 1985 by Leskelä et al. [18]. They utilized anhydrous zinc acetate (Zn(CH_3_COO)_2_) and H_2_O to grow thin films of ZnO on a soda glass plate substrate using atomic layer epitaxy. In the following years, many groups reported deposition of ZnO thin films by using different precursor combinations, such as Zn/H_2_O [19], Zn/O_2_ [20] ZnCl_2_/O_2_ [21], Zn(OAc)_2_/H_2_O [22], Zn(C_2_H_5_)_2_/H_2_O [23], Zn(C_2_H_5_)_2_/O_2_ [24], Zn(C_2_H_5_)_2_/O_2_ plasma [25], Zn(C_2_H_5_)_2_/N_2_O [26], and Zn(CH_3_)_2_/H_2_O [27] on flat substrates. Zn(C_2_H_5_)_2_/H_2_O is the most used precursor-reactant combination for deposition of ZnO on substrates, owing to diethylzinc’s high vapor pressure and high reactivity. Yousfi et al. [28] studied the growth behavior of ZnO thin films via atomic layer epitaxy using an in-situ quartz crystal microbalance. They showed the effect of temperature (RT to 200 °C) and pulse time on ZnO deposition through analysis of nucleation and coalescence effects. Libera et al. [29] reported the deposition of conformal ZnO coatings on silica with a specific surface area of 100 m²/g. The ZnO ALD was carried out in a viscous flow reactor on 1 g batches of powder. However, the homogeneity of ZnO deposition was confirmed on the micrometer scale, and the authors indicated that chemical vapor deposition of Zn metal occurred within their studied temperature range (150–200 °C).

There are several examples for the application of ZnO ALD in the field of catalysis research. Lei et al. [30] promoted Pt nanoparticles on Al_2_O_3_ support using ZnO ALD for aqueous phase reforming of 1-propanol. Regarding analysis of ALD processes, this study focused on Pt ALD but lacked details regarding ZnO ALD. Gong et al. [31] modified microporous ZSM-5 and Y type zeolites via ALD of ZnO for catalytic conversion of propane. Wang et al. [32] reported the growth modes of ZnO in the porous framework of HZSM-5 type zeolites. However, in both studies, growth of nanosized ZnO particles on the exoskeleton of the zeolites within just 1–2 ALD cycles was reported. Such a low cycle number would not result in formation of nanocrystalline particles, if the involved process was actually ALD. It is a common practice in the ALD community to demonstrate the self-limiting feature of the ALD process using a quartz crystal microbalance (flat substrate) and replicate the conditions accordingly for ALD on powders. However, ALD on powders has different challenges compared to ALD on flat substrates. Porous powders have a high specific surface area, which require longer dosing times for ALD processes and need to overcome pore diffusion problems for many bulky precursors [33]. Due to the porosity and irregular shape, precursors can sterically hinder other precursor molecules from coating the complete area, resulting in low deposition rates compared to flat substrates. Strempel et al. [34] designed an ALD set-up capable of measuring the self-limiting nature of an ALD process on powders using an in-situ magnetic suspension balance. The ALD process of TMA/H_2_O to deposit alumina on silica was used as proof of principle [35]. The same method was also used to investigate the complex nature of alucone growth during molecular layer deposition [36]. Here, we will utilize the same technique of in-situ thermogravimetry to provide insights into growth and reaction mechanisms of ZnO ALD on a porous powder.

In summary, high surface area ZnO was synthesized using atomic layer deposition. ALD of ZnO was carried out using alternating exposure of diethyl zinc (DEZn) and water (H_2_O) to a high surface area silica support at 80 °C and atmospheric pressure. The growth behavior of ZnO ALD on SiO_2_ was studied by using a magnetic suspension balance. A thin conformal layer of ZnO was formed on the high surface area silica using varying number of ALD cycles. The ALD process was later scaled up in a fixed bed (~30 mL volume).

## 2. Materials and Methods

Silica powder (SiO_2_, high-purity grade, ≥99%, Davisil Grade 636, average pore size 60 Å, 35–60 mesh particle size, Sigma-Aldrich, St. Louis, Mo, USA), specific surface area 506 m^2^/g) was used as substrate for atomic layer deposition. Diethyl zinc (Zn(C_2_H_5_)_2_, DEZn, elec. gr., 99.999% Zn, Sterm Chemicals GmbH, Kehl, Germany) and water (H_2_O, CHROMASOLV^®^, for HPLC, Riedel-de Haën/Honeywell Specialty Chemicals Seelze GmbH, Seelze, Germany) served as precursors and were used without further purification. High purity N_2_, Ar, and He (99.999%) were used as carrier and purging gases.

### 2.1. Atomic Layer Deposition of ZnO on SiO_2_: In-Situ Thermogravimetry

The growth behavior of ZnO on mesoporous SiO_2_ was studied in a home build set-up. The detailed description of the set-up is given elsewhere [34]. The ALD process was carried out under continuous total gas flow of 50 mL/min at atmospheric pressure and a deposition temperature of 80 °C. The diethyl-zinc (DEZn) saturator was heated to 50 °C, while the water saturator was kept at room temperature. Precursor and reactant were sequentially introduced into the fixed bed reactor in a continuous flow of 50 mL/min carrier gas. The sequence used was DEZn/N_2_-Ar purge-H_2_O/He–Ar purge. The reactants were dosed onto the substrate until no further mass gain was observed by the magnetic suspension balance. Valve switching between the ALD sequences was done manually. In total, 1, 2, and 3 cycles of ZnO ALD were carried out on SiO_2_.

### 2.2. Scale up of Atomic Layer Deposition of ZnO on SiO_2_

The ALD process of ZnO on SiO_2_ was scaled up in a fixed bed reactor made of quartz. The ALD process was carried out under a continuous total gas flow of 200 mL/min at atmospheric pressure and a deposition temperature of 80 °C. The diethyl-zinc (DEZn) saturator was heated to 50 °C, while the water saturator was kept at room temperature. Precursor and reactant in this ALD experiment were sequentially introduced into the reactor with continuous flow of 200 mL/min carrier gas. The sequence used was DEZn/N_2_-Ar purge-H_2_O/He–Ar purge. Typical half cycle times were determined based on online mass spectrometry. The precursor was dosed to the reactor until a breakthrough signal (*m*/*z*: 93) for DEZn and (*m*/*z*: 18) for H_2_O was observed. Valve switching between the ALD sequences was done manually. In total, 1, 2, and 3 cycles of ZnO ALD were carried out on SiO_2_.

### 2.3. Characterization of Materials

Nitrogen sorption analysis was performed at 77 K using a Quadrasorb SI (Quantachrome GmbH & Co. KG Odelzhausen, Germany), and the samples were degassed at 120 °C for 12 h prior to measurements. The surface area was determined using the Brunauer–Emmett–Teller (BET) method, and the corresponding pore size distribution was obtained by the adsorption branch isotherm using the BJH method (Barrett, Joyner, and Halenda). X-ray powder diffraction (XRD) patterns were recorded on a Bruker D8 diffractometer (Bruker AXS GmbH, Kalrsuhe, Germany) with Cu Kα1 radiation (λ = 0.154 nm) equipped with scintillation counter. TEM and energy dispersive X-ray spectroscopy (EDX) analyses were performed with a TECNAI G^2^20 S-TWIN electron microscope (FEI, Hillsboro, OR, USA) operated at 200 kV, equipped with an EDAX EDX system (Si(Li) SUTW detector, energy resolution of 136 eV for MnKα) X-ray fluorescence spectroscopy (XRF) was performed in a Bruker S4 Pioneer X-ray spectrometer (Bruker AXS GmbH, Kalrsuhe, Germany). Samples were prepared by melting pellets with a ratio of 100 mg sample with 8.9 g of Li_2_B_4_O_7_. The surface density of OH groups on the used SiO_2_ was determined via a titration method described elsewhere [37]. A NMR tube was charged with 30 mg of SiO_2_, 10 mg of ferrocene, and 25 mg of Mg(CH_2_Ph)_2•_2(THF) inside the inert atmosphere of a glovebox. Mg(CH_2_Ph)_2•_2(THF) was synthesized according to literature [38]. The solid mixture was suspended in 0.5 mL of benzen-d_6_. The NMR tube was sealed and shaken for several minutes to let the reagents react with SiO_2_. ^1^H-NMR spectra were recorded via a Bruker Avance II 200 MHz spectrometer (Bruker BioSpin MRI GmbH, Ettlingen, Germany). The total number of OH sites on the SiO_2_ was determined by calculating the number of moles of toluene produced (signal for methyl group at 2.1 ppm) using ferrocene as internal standard.

## 3. Results

### 3.1. In-Situ Thermogravimetry

**Figure 1:**
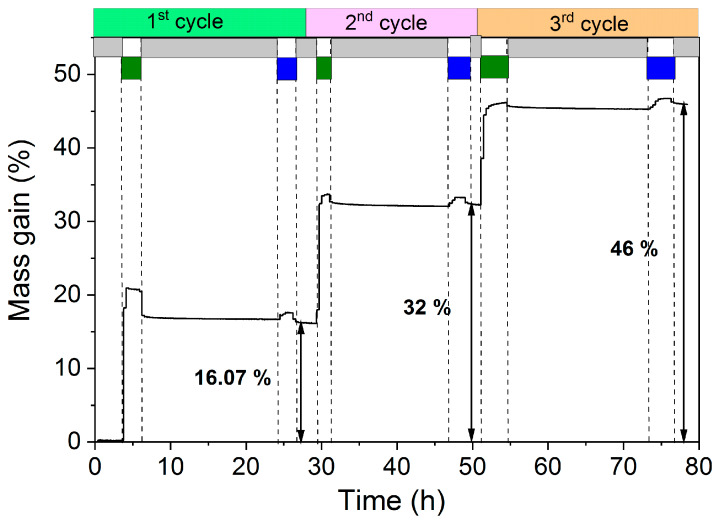
Mass gain as function of time during deposition of ZnO on SiO_2_ at 80 °C, N_2_ and He was used as carrier gas for diethyl zinc (DEZn) and H_2_O respectively. DEZn precursor was saturated at 50 °C, while H_2_O was saturated at room temperature. The flows for all gases were kept at 50 mL/min.

The high surface area silica was successfully coated with conformal layers of ZnO via an atomic layer deposition process of DEZn/H_2_O at 80 °C and atmospheric pressure. An in-situ thermogravimetric study using a magnetic suspension balance was carried out to study the growth behavior of ZnO on silica. 200 mg of silica powder was positioned in the magnetic suspension balance, and 1, 2, and 3 ALD cycles of ZnO were conducted via sequential exposure of DEZn and H_2_O. The gravimetric data of the first 3 cycles are shown in Figure 1. In the first step, the balance was purged with Ar to remove any physisorbed water, and then the balance was tared to zero. Afterwards, when DEZn/N_2_ was fed into the reactor, a sudden increase in mass gain was observed. This increase in the mass corresponds to the chemisorption of DEZn on hydroxyl terminated SiO_2_ with C_2_H_6_ as a leaving group. The self-limiting nature of the ALD process was observed from the gravimetric data. When all hydroxyl groups were saturated with chemisorbed monoethylzinc, no excessive mass gain was observed even after prolonged dosing of DEZn. In the third step, the balance was purged with Ar gas to remove any physisorbed DEZn from the substrate. Finally, H_2_O/He was introduced to the balance. Water reacts with terminal ethyl groups of the chemisorbed monoethyl zinc to generate a new hydroxyl group with ethane as a leaving molecule. No mass change or little decrease in mass gain was observed at this stage. This is due to the addition of hydroxyl groups (+17 g/mol) to the chemisorbed species and the removal of ethyl ligands (−29 g/mol). Note that we use mass gain values during Ar purge as measurement points to overcome the effect of buoyancy due to the use of different gases to introduce the precursor and reactant. Overall, 16.07 wt.% mass gain was observed on the SiO_2_ after the first ALD cycle of ZnO, followed by 32 wt.% after the second ALD cycle, and 46 wt.% after the third ALD cycle. The gravimetric data can be analyzed more precisely by looking at the individual uptake of each half cycle and associating the data to propose a possible reaction mechanism for the surface reactions.

**Figure 2:**
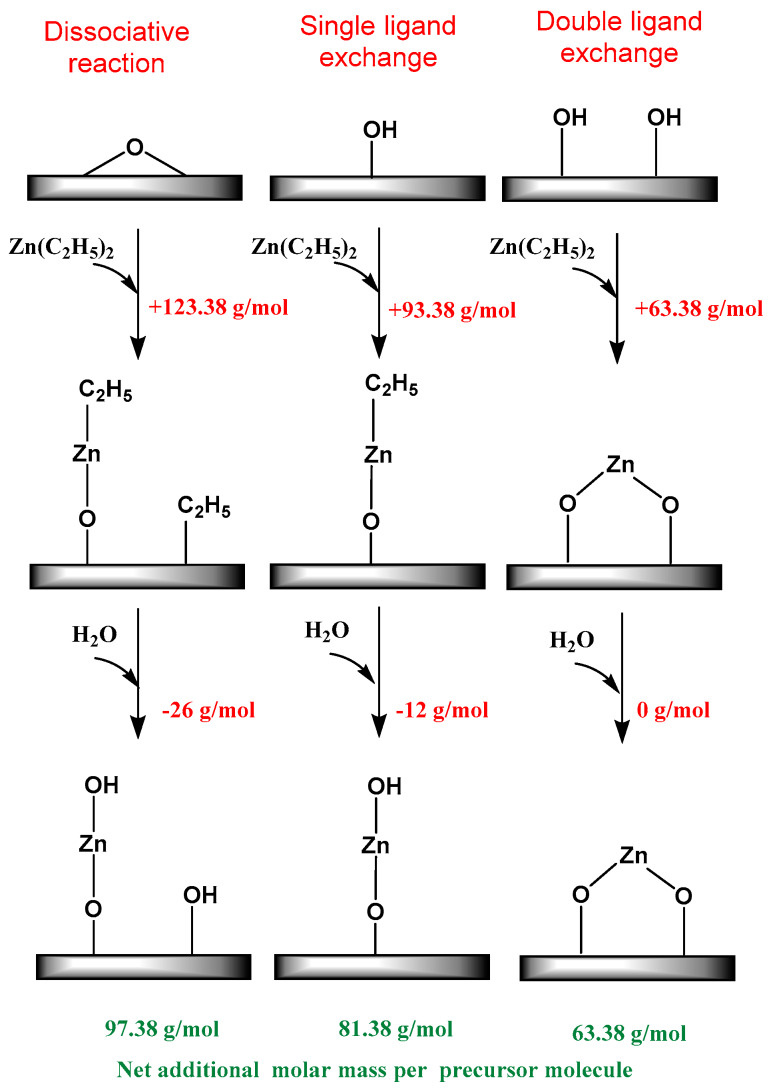
Postulated surface reaction happening during the ALD process of DEZn and H_2_O. The reaction mechanisms are classified in three types, namely dissociative reaction, single ligand exchange, and double ligand exchange. The mass change associated with each step is given in the figure.

DEZn can react with surface hydroxyl groups (|−OH) by either dissociative reaction or single or double ligand exchange as shown in Figure 2. The associative and dissociative type of surface reactions is neglected for sake of simplicity.

(1) $$\left|-{\left(\mathrm{OH}\right)}_{x}+Zn{\left(C_{2}H_{5}\right)}_{2}\;\to \right|-O_{x}-Zn{\left(C_{2}H_{5}\right)}_{2-x}+xC_{2}H_{6}$$

(2) $$\left|-O_{x}-Zn{\left(C_{2}H_{5}\right)}_{2-x}+H_{2}O\;\to \right|-\mathrm{OZn}-{\left(OH\right)}_{x}+\left(2-x\right)C_{2}H_{6}$$

Here, x is between 0 and 2, which corresponds the number of the hydroxyl group per Zn site. In the equation above, if x = 1, then the surface reaction is a single ligand exchange reaction, where DEZn will chemisorb on a single hydroxyl group to form a monoethyl zinc after the first half cycle. This monoethyl zinc is later hydroxylated to form a surface hydroxyl group while releasing ethane. Based on the calculations, the single ligand exchange will contribute to additional 81.5 g/mol (Pre) and a double ligand exchange will contribute to the additional 63.5 g/mol (Pre). In our experiments, the overall added molar mass was calculated using the mass gain and Zn content, which was measured ex-situ after the experiment using XRF. The results from the balance yields 82.4 g/mol (Pre) after the first cycle, 81.6 g/mol (Pre) after the second cycle and 80.6 g/mol (Pre) after the third cycle. The molar mass of precursor chemisorbed on the surface was calculated by dividing the total mass gain obtained from the balance to moles of zinc in the sample. The moles of zinc in the sample can be calculated by the experimental Zn content obtained via XRF. The experimental results indicated that the growth of ZnO on mesoporous SiO_2_ is clearly driven by the single ligand exchange mechanism. Single ligand exchange is an ideal surface reaction that can occur at the surface of the substrate. However, the DEZn molecule can also react via alternative pathways on irregular porous surfaces where presence of micro-porosity can yield steric hindrance. Our results and calculations were in total agreement with a comprehensive density functional theory study carried out by Weckmann et al. [39] for surface reactions of DEZn. Weckmann et al. found that DEZn quickly reacts with the surface hydroxyl groups to form a monoethylzinc-saturated surface. At low deposition temperatures, the reaction stops at this point. However, at higher deposition temperatures, monoethylzinc will react with further precursor molecules leading to a higher deposition rate and CVD-like growth. This data was in agreement with the ideal single ligand exchange mechanism derived from our mass gain data. We also observed a slightly lower mass change associated with the second step (H_2_O) than expected; however, this data was also in agreement with ab initio calculations of Weckmann et al. [40] where they demonstrated the higher energy barrier in surface reaction between water and ethyl groups, where even after water cycles, some ethyl groups might persist on the surface.

### 3.2. Scale-up of the ALD Process in a Fixed Bed Reactor

**Figure 3:**
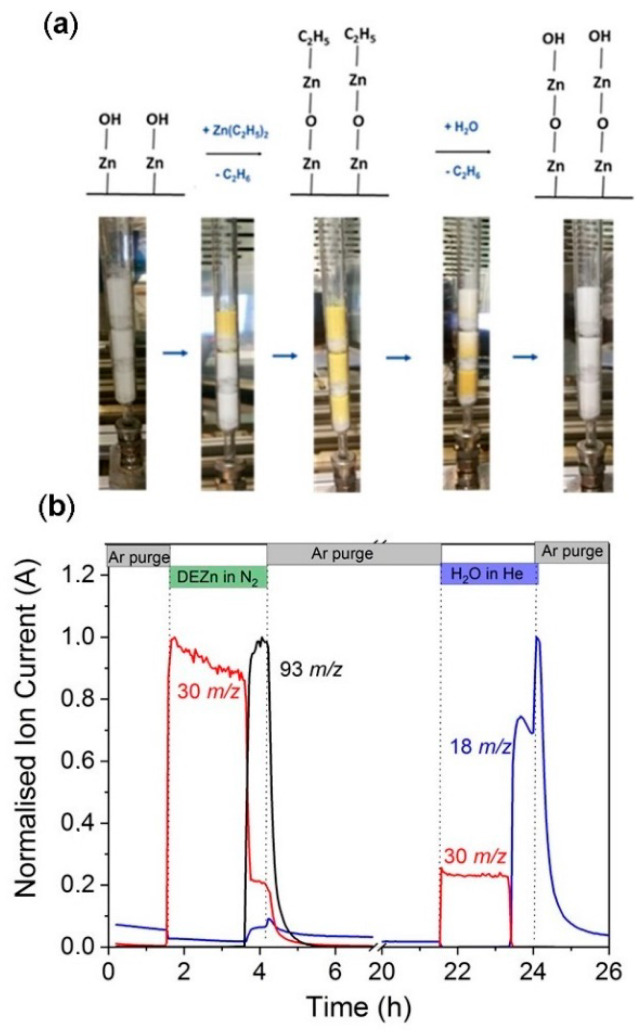
Scale-up of ZnO ALD on SiO_2_ in a fixed bed reactor. (**a**) The different steps highlight two half cycles of the ALD process proceeded with sequential and simultaneous color change of ZnO/SiO_2_ from yellow to white and (**b**) online mass spectrometry showing breakthrough curves for one ZnO ALD cycle on SiO_2_. Normalized ion current over time for following mass to charge ratios: 18 (H_2_O^+^), 30 (C_2_H_6_^+^), and 93 (Zn(C_2_H_5_)^+^). Note that there is a time break on the X axis during the Ar purge from 7 to 20 h.

Studying the ALD process in the magnetic suspension balance not only provided deep insights into the saturation behavior of precursors and reactants but also an understanding of the underlying surface reaction mechanism. However, such experiments can only coat a 1 mL volume of material. To be able to coat larger volumes of powder for the catalyst synthesis, we scaled up the ALD of ZnO process in the fixed bed reactor with a maximum volume of ~30 mL. A quartz tube was used as a reactor, and SiO_2_ powder was packed in the reactor as shown in Figure 3a. After the first full ZnO ALD cycle, we observed a sequential color change from white to yellow during DEZn dosing. The color changed chromatographically along the flow of DEZn in the reactor. The change in color is an indication of the reaction of DEZn with hydroxyl-terminated groups on 1c-ZnO/SiO_2_. The yellow color was reversed to white upon dosing of H_2_O. Zinc oxide naturally occurs as a white powder; however, upon heating at a high temperature, ZnO loses oxygen and forms a nonstoichiometric oxygen deficient ZnO structure, which shows a yellow color [41]. The ALD was carried out at 80 °C, which is a low temperature to form oxygen deficiency. However, during the second ALD cycle the chemisorption of DEZn on hydroxyl-terminated ZnO/SiO_2_ forms an oxygen deficient ZnO structure. After the water dosing, the surface is again populated with hydroxyl groups, which neutralizes the oxygen deficiency within the surface ZnO films; hence, the color of ZnO reverses back to white. This visible effect again proves the self-limiting and sequential nature of ZnO ALD in the fixed bed reactor.

The self-limiting behavior of each ALD half cycle was monitored using online mass spectrometry; the breakthrough curves for the first cycle are shown in Figure 3b. The breakthrough curve in mass spectrometry developed simultaneously with the color change observed in the fixed bed reactor. During the first half cycle, protonated ethane (*m*/*z* = 30) was observed upon dosing of DEZn to the reactor, indicating chemisorption of DEZn to the surface hydroxyl groups. The protonated ethane (*m*/*z* = 30) signal decreased with increase in the Zn(C_2_H_5_) + signal (*m*/*z* = 93) indicating the chemisorption of DEZn on all surface hydroxyl groups. The chromatographic color change from white to yellow was observed at exactly the same time as the breakthrough of the Zn(C_2_H_5_) + signal (*m*/*z* = 93) emerged. Note that background of the water (*m*/*z* = 18) signal can be seen throughout the first half cycle and the slight increase in the water (*m*/*z* = 18) signal at the breakthrough curve is due to pressure change in the mass spectrometer chamber. In the second half cycle, the emergence of a protonated ethane (*m*/*z* = 30) signal indicates the surface reaction of monoethyl zinc with water. The breakthrough curve for water (*m*/*z* = 18) emerged upon complete exchange of ethyl groups by hydroxyl groups. The visual effect of this step was seen in the fixed bed by color change from yellow to white.

### 3.3. Characterization of ALD coated ZnO/SiO_2_ Samples

**Figure 4:**
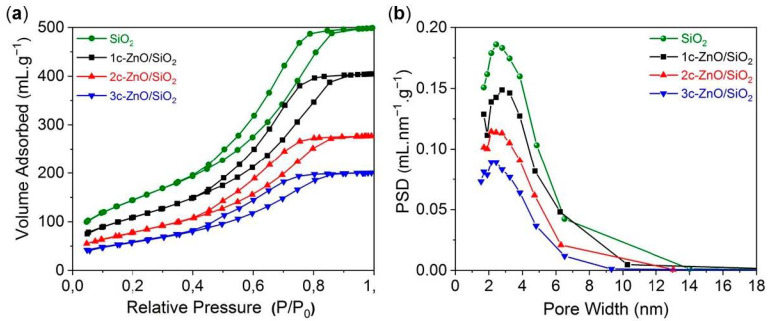
(**a**) N_2_ sorption isotherms and (**b**) pore size distribution calculated by adsorption branch of isotherm using BJH method of ZnO coated SiO_2_ at 80 °C.

The synthesized ZnO/SiO_2_ samples were characterized using N_2_ sorption analysis. N_2_ sorption isotherms and pore size distribution curves are shown in Figure 4a,b. All samples showed similar H_1_ type hysteresis loops indicating conformal coating of ZnO in the mesopores of SiO_2_. The volume of N_2_ adsorbed decreased as a function of the ALD cycle number thereby decreasing the total pore volume. As expected, the specific surface area of SiO_2_ decreased drastically after 1, 2, and 3 cycles of ZnO ALD. While the pristine SiO_2_ had a specific surface area of 505 m²/g, after 3 ALD cycles, the specific surface area changed to 213 m²/g. To the best of our knowledge, this surface area is higher compared to many other reported ZnO nanostructures. If we consider the deposition rate of ZnO from literature at 80 °C to be ~0.1 nm/cycle [42] and the bulk density of ZnO to be 5.61 g/cm³, we can calculate the expected mass gain of ZnO after 3 cycles as follows:

Expected mass gain of ZnO = 3 cycles × 0.1 nm/cycle × 5.61 g/cm³ × 505 m²/g = 0.849 g (ZnO)/g (SiO_2_).

This corresponds to the mass gain of 45.9 wt.%. The experimental mass gain obtained from the balance is 46 wt.%, which is in complete agreement with the theoretical calculations and assumptions. The pore size distribution curves for the ZnO/SiO_2_ samples are shown in Figure 4b. The pore size distribution was calculated using the BJH method with assumption of regular and cylindrical pores. The average pore size of SiO_2_ decreased from 4.8 nm to 4.2 nm after 3 cycles of ZnO ALD. This is an indication of ideal ZnO growth on SiO_2_ surface. The results were in agreement with assumption of 0.1 nm/cycle. All physiochemical analyses of ALD-prepared ZnO/SiO_2_ samples can be seen in Table 1.

A titration method was used to quantify the initial surface -OH group density of the used SiO_2_ powder. The recorded ^1^H-NMR spectrum used for quantification is provided in Supplementary Material (Figure S1). The surface -OH group coverage on mesoporous SiO_2_ was found to be ~3.5 OH groups/nm². The calculated surface coverage of Zn atoms after the first ALD cycle was ~2.8 Zn _atoms_/nm². The fact that not all –OH surface groups (only ~80%) reacted with DEZ can be rationalized by steric hindrance.

**Figure 5:**
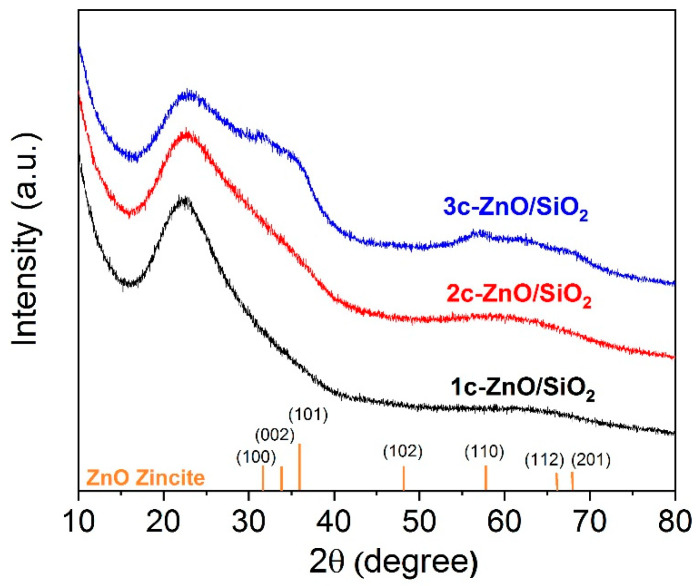
XRD patterns of 1, 2, and 3 cycles of ZnO coated SiO_2_ at 80 °C.

X-ray diffraction analyses of pristine and SiO_2_ coated with ZnO are shown in Figure 5. The broad peak at 2θ = 21.8° is associated with amorphous SiO_2_. After the first and second ALD cycle, the diffractograms show no indication of any crystalline phase, i.e., the deposited ZnO is X-ray amorphous. After 3 ZnO ALD cycles, a broad peak appears between 2θ = 30° and 40° and another broad peak appears at 2θ = 57°. These peaks are too broad for any kind of assignment but can be rationalized by the presence of nanocrystalline ZnO domains of hexagonal zincite (PDF 36-1451). It is remarkable that even after deposition of 45 wt.% ZnO no crystallinity is observed. This fits in well with our goal of depositing a thin and homogeneous layer of ZnO. A thin layer of less than 1 nm thickness should not give XRD reflections, as the number of ordered layers is well below that needed for diffraction and interference of X-rays. Libera et al. [29] described the presence of a Zn metal peak even after 1 ALD cycle, and this peak became more prominent with increasing deposition temperature from 150 °C to 200 °C. The presence of metallic Zn is an indication that the process is changing towards CVD. An ALD process relies on sequential reaction (chemisorption) while CVD is driven by thermal decomposition of precursors at the surface. Many studies deposit ZnO at higher temperatures to achieve high growth rates but often the process tends to be a mixture of ALD and CVD. In our study, we deposited ZnO at 80 °C while achieving ZnO growth and preventing formation of metallic Zn through parasitic chemical vapor deposition.

**Figure 6:**
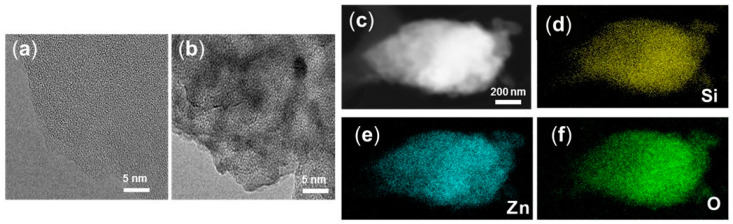
HRTEM images of (**a**) SiO_2_ (**b**) 3c-ZnO/SiO_2_; STEM image of (**c**) 3c-ZnO/SiO_2_ and EDX mapping of (**d**) Si, (**e**) Zn, (**f**) O.

The HRTEM and STEM-EDX mapping images of 3c-ZnO/SiO_2_ are shown in Figure 6. The overall structure and morphology of the material is similar to that of uncoated mesoporous silica. However, after 3 cycles of ZnO ALD on SiO_2_, lattice fringes for deposited ZnO can be seen in the HRTEM images. The presence of these lattice fringes indicates the nano-crystallinity present in the ZnO films. We did not detect larger clusters or agglomerates of ZnO particles or presence of metallic Zn on SiO_2_, indicating homogenous deposition of the ZnO films during the ALD process. The STEM-EDX mapping showed exact overlapping Zn and Si signals, indicating complete coverage of bulk SiO_2_ with very thin layers of ZnO after 3 ALD cycles. Atomic layer deposition proved to be an effective way to coat the high surface area powder and the process could be easily scaled up. Using ALD as a tool to synthesize high surface area ZnO with SiO_2_ as bulk material opens up many possibilities in heterogeneous and photocatalysis for precise design of ZnO based materials.

## 4. Conclusions

To the best of our knowledge, we showed for the first time directly the self-limiting nature of ZnO ALD on porous powders using in-situ thermogravimetry. We successfully synthesized thin layers of ZnO on SiO_2_ using 1, 2, and 3 cycles of ALD in a fixed bed reactor. We provided deep insights into the saturation behavior of diethylzinc on powder and postulated a surface reaction mechanism based on the mass gain for the individual half cycles. The total mass gain measured in situ fits well to the Zn content measured via XRF. We showed that the ALD of ZnO on high surface area silica proceeds via an ideal single ligand exchange mechanism. ALD proved to be an efficient method to achieve conformal coating on highly porous material such as silica (Davisil grade 636). We complemented the mechanistic study on ZnO ALD by transferring the process to a 30 mL volume fixed bed reactor. To the best of our knowledge, we report for the first time the visual color change occurring during half cycles of the ZnO ALD process on powders. In summary, this study provides in-depth understanding of the ZnO ALD on porous powders. The knowledge of the underlying mechanisms of the ALD process will aid in precise design and modification of catalytic materials.

## Figures and Tables

**Table 1:** Physiochemical analysis of the ZnO ALD coated mesoporous high surface area silica.

Sample Name	ALD Cycles	Specific Surface Area (m²/g)	Average Pore Volume (cc/g)	ZnO Content (XRF) (wt. %)	Total Mass Gain from Balance (wt.%)
SiO_2_	0	505	0.75	0	0
1c-ZnO/SiO_2_	1	400	0.56	15.7	16
2c-ZnO/SiO_2_	2	289	0.43	31.8	32
3c-ZnO/SiO_2_	3	213	0.31	45.1	46

## Supplementary Materials

The following are available online at https://www.mdpi.com/2079-4991/10/5/981/s1, Figure S1: Recorded 1H-NMR spectrum of the reaction mixture of the titration experiment.
