# Supplementary material for: Atomic Layer Deposition of ZnO on Mesoporous Silica: Insights into Growth Behavior of ZnO via In-Situ Thermogravimetric Analysis
Source: Nanomaterials (Basel). 2020 May 20;10(5):981. doi: 10.3390/nano10050981 (PMC7279530; doi:10.3390/nano10050981)
Supplement: Supplementary file 1 [file nanomaterials-10-00981-s001.pdf]

nanomaterials

Supplementary Materials

Atomic Layer Deposition of ZnO on Mesoporous Silica: Insights into Growth Behavior of ZnO via In-Situ Thermogravimetric Analysis

Piyush Ingale [1], Kristian Knemeyer [1], Mar Piernavieja Hermida [1], Raoul Naumann d'Alnoncourt [1,*], Arne Thomas [2] and Frank Rosowski [1,3]

[1] BasCat—UniCat BASF JointLab, Technische Universität Berlin, Hardenbergstraße 36, 10623 Berlin, Germany; p.ingale@bascat.tu-berlin.de (P.I.); k.knemeyer@bascat.tu-berlin.de (K.K.); m.piernaviejahermida@tu-berlin.de (M.P.H.); frank.rosowski@basf.com (F.R.)

[2] Institut für Chemie, Technische Universität Berlin, 10623 Berlin, Germany; arne.thomas@tu-berlin.de

[3] Process Research and Chemical Engineering, BASF SE, 67056 Ludwigshafen, Germany

* Correspondence: r.naumann@bascat.tu-berlin.de

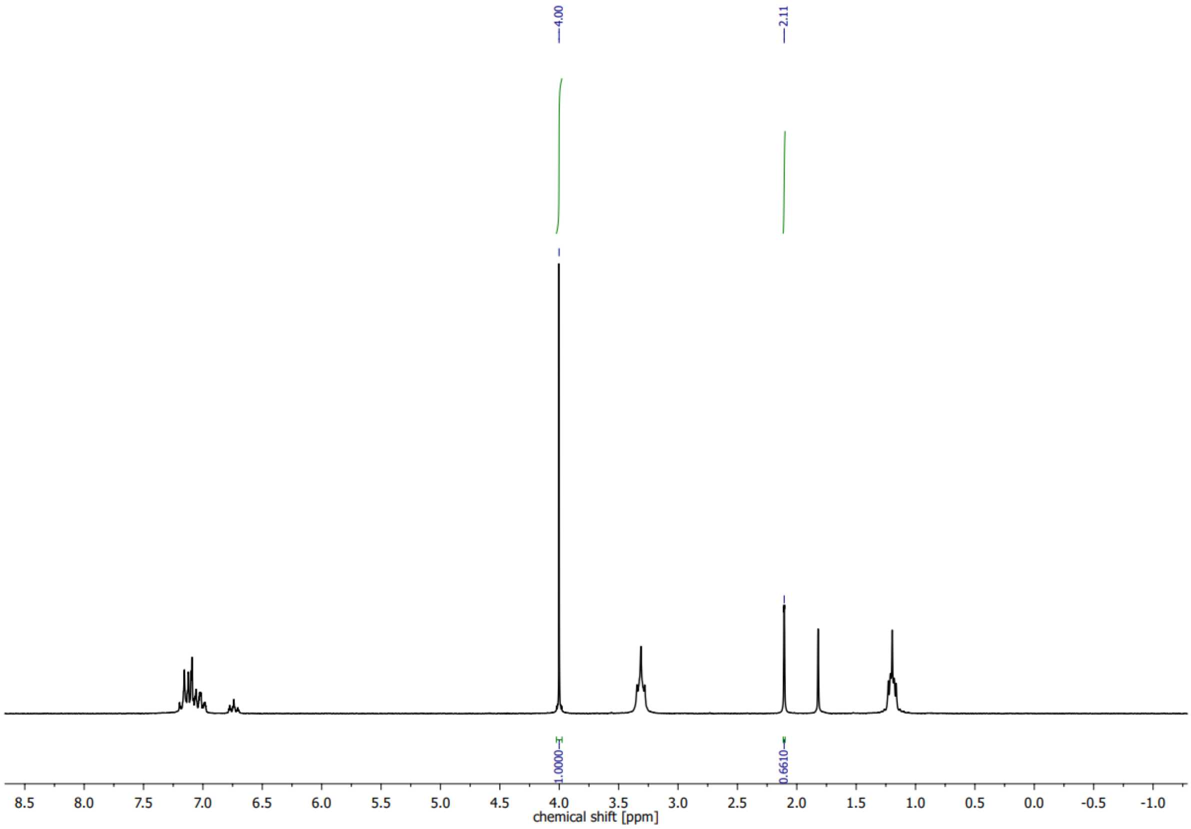

Figure S1. Recorded 1H-NMR spectrum of the reaction mixture of the titration experiment. The amount of formed toluene is related to the formed peak at 2.11 ppm compared to the used reference ferrocene.
